# Postural balance ability and vertical jumping performance in female veteran volleyball athletes and non-athletes

**DOI:** 10.3389/fspor.2023.1109488

**Published:** 2023-05-11

**Authors:** Maria-Elissavet Nikolaidou, Konstantinos Sotiropoulos, Karolina Barzouka

**Affiliations:** School of Physical Education and Sport Science, Department of Physical Education and Sport Science, National and Kapodistrian University of Athens, Athens, Greece

**Keywords:** long-term training, elite, team sports, postural performance, biomechanics, countermovement jump

## Abstract

Lifetime participation in sports is associated with improved components of physical conditioning. The main purpose was to cross-sectionally investigate postural balance and vertical jumping performance in athletes with different histories of sports participation and secondarily to examine the restriction of vision on balance ability. A final aim was to investigate possible associations between balance and jumping performance. We hypothesized higher balance and jumping performance in active veteran volleyball athletes compared to retired athletes and non-athletes, suggesting a positive effect of continuous systematic training in active veteran athletes. We also hypothesized greater negative effect of vision removal on balance in the veteran compared to non-athletes due to athletes' stronger reliance on visual information. Eighty-one healthy middle-aged women (mean (standard deviation) 50 (5) years) were assigned to three experimental groups, a retired (*n* = 39, recreationally active former athletes), an active (*n* = 27, training 2days/week x 1.5 h/session) veteran volleyball athletes' and a control group (*n* = 15, sedentary participants). Participants completed an assessment of single-legged quiet stance trials with either left **or** right leg with eyes open while standing barefoot on a force plate and two-legged trials with both eyes open or closed. They also executed a protocol of countermovement jumps. Statistical analyses included univariate and full factorial ANOVAs with group and vision as fixed and repeated-measures factors and simple linear regression analysis. In the single-legged balance task, solely the mediolateral sway range was greater for the active (*p* < 0.001) and retired athletes (*p* < 0.001) compared to non-athletes, whereas in the two-legged stance, no differences among groups were found (*p* > 0.05). Restriction of vision deteriorated balance performance similarly in the three groups as a significant vision effect was found for path length (*p* < 0.001), anteroposterior (*p* < 0.001) and mediolateral sway (*p* < 0.05). The active and retired athletes had significantly (*p* < 0.001) greater height, mean and maximal power in countermovement jump compared to non-athletes. Results showed weak associations (average *R*^2^ = 9.5%) of balance with jumping performance only in the veteran volleyball athletes' group. Overall, the findings showed that retired volleyball athletes exhibited similar balance ability and vertical jumping performance as the active ones, suggesting a positive impact of prior experience in systematic training.

## 1. Introduction

Volleyball is a demanding team sport consisting of complex technical-coordinative variations of movements (1). Typically, the majority of scoring points relies on the efficiency of actions that are performed airborne (i.e., spike, block and serve) (2). Moreover, control of the position and orientation of intersegmental movements during the approach phase and at take-off is required for maximal performance as well as for optimal landing during a spike jump (3, 4). It has also been stressed that the effectiveness of volleyball athletes depends on the postural stability and movement precision they exhibit during the game's non-airborne actions (i.e., set, reception, defense) (5, 6). During defense, for example, where spike velocities range from ∼50 to 112 km/h for top-level male and female athletes (7), it is important that athletes are capable of optimally aligning their body position in relation to the anticipated ball trajectory and having control of their postural sway at the time of ball contact (8). Young male and female volleyball athletes were found to have a superior postural balance performance compared to age-matched non-athletes, whereas elite or top-level athletes exhibited higher postural skills when compared with athletes at lower competition levels (4, 5, 8–10).

Furthermore, the game's actions are typically executed at high-intensity within periods of short duration (i.e., 3–9 s) (11) and thus, volleyball places such demands on the neuromuscular system that target the development of high levels of muscle strength and power. During training and competition, a high amount of single- and two-legged jumps and landings is performed (12) and upon examining the physical and physiological attributes it was observed that volleyball players of better performing teams had higher vertical jumping performance (13, 14). Berriel et al. (15) found that attack jump height correlated strongly with countermovement jump (CMJ) height, whereas increases in depth-jump ability were strongly associated with increases in CMJ performance over a 12-month training period in elite male volleyball players (16). Therefore, training and enhancing the vertical jumping abilities of athletes is recognized as an important factor influencing performance in volleyball (17, 18).

The popularity of volleyball does not appear to attenuate with age since middle-aged or older veteran athletes train regularly and compete in local or even international tournaments (19). Lifetime participation in elite-level sports is associated with improved components of physical conditioning (e.g., explosive muscle strength, balance) for former male athletes (20–23), whereas respective evidence on former female athletes appears comparatively limited. Static and dynamic balance has been examined in former elite female athletes and non-athletes over 50 years of age with a sport background mainly in athletics (24–26). To the best of the authors’ knowledge, one cross-sectional study until now has investigated balance in female active and retired veteran volleyball athletes (27). Results showed a superior static balance performance (i.e., lesser center of pressure sway amplitude) for the younger (<51 years) active veteran volleyball athletes compared to age-matched retired as well as older (≥51 years) active and retired veteran volleyball athletes (27). Further, whereas vertical jumping performance is a key component of a successful outcome in team sports, and earlier studies have shown that former elite male power-trained athletes have greater jump height in comparison with endurance-trained athletes and age-matched controls (20–22), there is no such respective evidence for former female athletes. Thus, the novelty of the study resides in examining the effect of history of sport participation in veteran female athletes from team sports.

The first aim of the study was to investigate the postural balance ability and vertical jumping performance in top-level female active and retired veteran volleyball athletes compared with a group of age-matched non-athletes. We hypothesized a higher postural balance ability as determined by lesser sway amplitude and a higher vertical jumping performance for the female active veteran volleyball athletes compared with the non-athletes. Similarly, we hypothesized higher balance and jumping performance for the retired veteran volleyball athletes compared with the non-athletes due to prior experience in systematic training in the former. Regarding the previously documented adaptations (20–26) in muscle strength and power as well as in balance as a result of prolonged sport participation, the second hypothesis was that the continuous participation in systematic training would have a positive impact on balance and jumping performance for the active veteran volleyball athletes compared with the retired athletes. Vision provides valuable information in ensuring an accurate perception and representation of body position and its movement with the environment (28). Athletes in team sports are considered to rely strongly on constant visual feedback to determine their interaction with their teammates and opponents under the effect of constant movement of the ball within the game field (9). The second aim of the study was to examine if visual restriction impacts the veteran volleyball athletes and non-athletes differently in a quiet stance balance test. We hypothesized greater balance deterioration (i.e., greater sway amplitude) due to visual restriction in both the active and retired veteran volleyball athletes than the non-athletes, indicating the superior contribution of the visual channel in postural balance performance of the veteran volleyball athletes. Adequate postural control during the take-off phase of jump in volleyball (3, 4) would create favourable conditions for the ensuing jumping task and could be associated with higher jumping performance. Thus, the final aim of the study was to investigate the association between static postural balance and vertical jumping performance. We hypothesized that the higher balance ability in the active and retired veteran volleyball athletes would favourably affect their jumping performance compared to non-athletes.

## 2. Methods

### 2.1. Participants

In this cross-sectional study, a total of 81 (mean (standard deviation), 50 (5) years) middle-aged women from five volleyball sport clubs and a group of sedentary women volunteered to participate. Inclusion criteria required that participants were healthy adults ≥45 years of age. Participants were asked about their medical and physical activity history and were excluded if they reported a history of neuromuscular diseases, musculoskeletal disorders, cardiovascular or severe systemic diseases, severe arthritis, or if they had been taking any medication for the above diseases in the last 6 months.

Participants were grouped into 3 experimental groups, specifically a non-athletes group (Control, *n* = 15), a group of retired veteran volleyball athletes (Retired, *n* = 39) and a group of active veteran volleyball athletes (Active, *n* = 27) (Table 1**)**. The control group was comprised of sedentary women who reported no physical activity history, as such being defined by a minimum of 30 min of exercise three times per week over the last 5 years (25). In the Retired volleyball group, 14 out of 39 athletes (36%) had systematically competed at the international level during their sport career, whereas the Active volleyball group consisted of athletes who had participated at the National A1—A2 League in the earlier stages of their sport career. Training experience for the Retired group amounted to 18 (8) years and for Active group at 15 (8) years, respectively. At the time of the study, the Retired group was engaged in recreational activities of low to moderate intensity 2–3 times/week, such as walking, light jogging, hiking and swimming, whereas the Active group trained at a frequency of two times/week for 1.5 h per session and competed at the Greek Veteran's Volleyball League with a weekly game. The study was approved by the Ethics Committee of the School of Physical Education and Sport Science, National and Kapodistrian University of Athens (approval number: 1163/12–02–2020), and all the participants gave their written informed consent in accordance with the Declaration of Helsinki.

**Table 1:** Anthropometric data for the three groups (mean (SD)).

	Control (*N* = 15)	Retired (*N* = 39)	Active (*N* = 27)
Age (years)	50.2 (4.4)	51.1 (5.3)	50.4 (4.4)
Body mass (kg)*	65.0 (8.7)^a^	73.3 (9.0)	68.3 (8.2)
Body height (m)*	1.62 (0.1)^a,c^	1.73 (0.1)	1.67 (0.1)^b,c^
Body mass index (kg/m^2^)	24.9 (2.8)	24.5 (3.1)	24.7 (3.1)

* Statistically significant group effect (*p* < 0.05).

^a^ Post hoc statistically significant (*p* < 0.05) differences between control and retired veteran volleyball athletes’ group (Retired).

^b^ Post hoc statistically significant (*p* < 0.05) differences between retired and active veteran volleyball athletes’ group (Active).

^c^ Post hoc statistically significant (*p* < 0.05) differences between control and Active group.

### 2.2. Assessment of postural stability performance

Balance performance was assessed in single-legged and two-legged quiet stance trials. The measuring device was located in the middle of a quiet, spacious room at an approximate distance of 2–3 m between the walls and the participants. During assessment of the single-legged trials, the participants had their eyes open, stood barefoot as motionless as possible with either their left or right leg on a vertical force plate (Wii, A/D converter, 24-bit resolution, 1,000 Hz, Biovision), and maintained a straight body posture with their arms hanging relaxed on their sides. They were instructed to fix their gaze on an imaginary point on the wall 2–3 m in front of them, while keeping their heads at a neutral position parallel to ground level. A randomized order was kept for the starting leg. For the two-legged quiet stance trials, the participants were instructed to keep their feet at hip-width apart and stand as motionless as possible with their eyes open, as described above, or eyes closed. In these trials, one researcher was always situated behind the participants for safety reasons.

Two successful trials were performed in the monopedal and bipedal quiet stance testing conditions in a randomized order. Duration of every trial was 20 s with 30 s of rest across trials and 1 min of rest between conditions. During the off-line analysis, the recorded center of pressure (CoP) data were filtered using a second bi-directional order digital low-pass Butterworth filter with a 15 Hz cut-off frequency and analyzed with MATLAB custom-made scripts (R2012a, 64 Bit; Mathworks, Natick, MA, United States). The data were analyzed from the 1st to the 16th second (*Δ*t = 15 s) of each 20 s trial time. Postural balance performance is typically assessed on the basis of the CoP displacement, which derived values represent the geometrical location of the reaction force vector on the platform during quiet standing (29) and was determined by the following parameters: (a) CoP path length, defined as the sum of Euclidean distances between adjacent measurement points, and (b) CoP sway range, defined as the range (i.e., from minimum to maximum) of the CoP values in the anteroposterior and mediolateral directions. Body height (30) and mass (31) may affect path length and sway range; therefore, we chose to normalize the determined CoP parameters to body height and mass and used the normalized values (% of body height per kilogram of body mass) for statistical analysis. For the two-legged stance trials, the average value of the two trials was used, whereas for the single-legged trials, no statistically significant differences were observed between sides (paired t test: *p* > 0.05) in the CoP parameters, the average value of the mean left and right leg trials was used for analysis.

### 2.3. Assessment of vertical jumping performance

To assess vertical jumping performance, the ground reaction force was recorded from a vertical force plate while participants completed a protocol of countermovement jumps (CMJs). Following a short familiarization protocol, which included 2–3 submaximal jumps with instructions focusing on starting from an erect position, no arm swing allowed, a depth of the downwards movement permitting an unobstructed push-off phase and full leg extension at the apex of the jump, the participants performed three successful two-legged CMJs. An audible command was first given and then participants performed a rapid stretch-shortening movement with self-determined countermovement. A rest interval of 2 min between familiarization and measurements and 30 s between jump trials was provided to minimize fatigue. Vertical center of mass velocity during the jump was calculated based on Equation 1 and jump height was then calculated using the vertical velocity of center of mass as shown in Equation 2. Positive mechanical power was calculated as the product of the vertical GRF and vertical center of mass velocity and was normalized to body mass. For the statistical analysis, the CMJ trial with the highest height achieved was chosen to determine the parameters of jump height, mean (Pmean) and maximal (Pmax) power.

Equation 1

$$V_{z(t)}=\overset{TO}{\underset{Start}{\int }}\frac{F_{z(t)}-mg}{m}\cdot dt$$

*V_z(t)_*: vertical velocity of the centre of mass over time.

*Fz(t)*: vertical ground reaction force over time.

*m*: mass of the participant.

*g*: acceleration of gravity.

*Start*: Start of centre of mass downwards movement, where *Fz* < body weight.

*TO*: Take off.

Equation 2

$$H=\frac{Vz_{TO}^{2}}{2g}$$

*H*: jump height.

*Vz_TO_*: vertical centre of mass velocity at *TO*.

### 2.4. Statistical analysis

All statistical analyses were performed using SPSS Statistics (Version 17.0). We checked for the normal distribution of the CoP data using the Kolmogorov–Smirnov test with Lilliefors correction. For the single-legged balance trials, the statistical testing of normality failed in two situations (Retired group: anteroposterior CoP range, *p* = 0.003; Active group: CoP path length, *p* = 0.006), whereas for the two-legged balance trials, the groups passed the majority of testing of normality in both the eyes open and eyes closed condition (except for Retired group: anteroposterior CoP range, *p* = 0.008 in the eyes closed condition). However, upon visual inspection with quantile-quantile (Q-Q) plots, the CoP data were normal with slight deviations. A one-way ANOVA with group (Control, Retired, Active) as fixed factor was performed to test for possible differences in the anthropometric, single-legged balance and vertical jumping performance parameters. A mixed ANOVA model was also performed with vision (open, closed eyes) as the within-subjects factor and group as the between-subjects factor on the two-legged balance performance outcome measures. A Bonferroni-corrected pairwise analysis was conducted in the case of a significant main effect or interaction effect between the factors of vision and group. Simple linear regression analysis was used to examine the association of two-legged balance performance with vertical jumping performance in each group. Goodness-of-fit was assessed by calculating the root mean square error (RMSE) and the coefficient of determination measure (*R*^2^ = 0–1.0) with larger values representing a better regression model. Specifically, values less than 0.5 indicate weak coefficient of determination, values between 0.5–0.75 indicate moderate, values between 0.75–0.9 indicate good and values greater than 0.9 excellent coefficient of determination, respectively. The level of significance for all the tests was set at a = 0.05. For the graphical representation of the balance performance outcomes, we used scatterplots with bars (mean ± standard error, which was calculated by dividing the standard deviation by the square root of number of participants for each group) depicting individual values, and scatterplots depicting the line of best fit and the 95% confidence interval bands were used to plot the linear regression analyses results.

## 3. Results

Age was not significantly different (*p* = 0.765) between the groups. There was a significant main effect of group for body mass (*F* = 5.607; *p* = 0.005) and *post hoc* comparisons showed that the Retired veteran volleyball athletes were significantly heavier (*p* = 0.007) than the control group (Table 1). A significant group main effect was also found for body height (*F* = 20.11; *p* < 0.001) and *post hoc* comparisons showed that the Retired veteran volleyball athletes were significantly taller compared to control (*p* < 0.001) and to the Active athletes' group (*p* < 0.001), whereas the Active group was significantly taller compared to control group (*p* = 0.018, Table 1). There were no significant differences between groups for body mass index (*p* = 0.895, Table 1).

**Figure 1:**
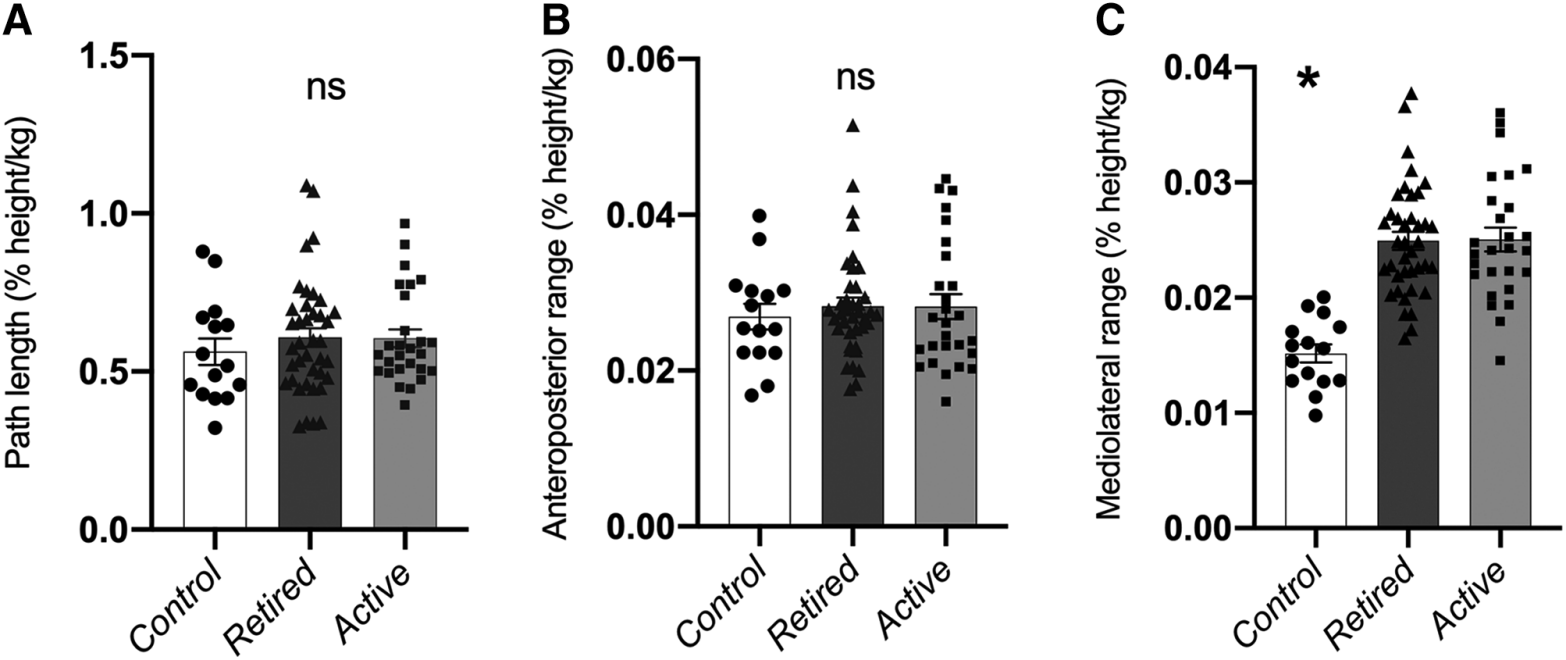
Normalized path length (**A**), CoP range in anteroposterior (**B**) and CoP range in mediolateral direction (**C**) in the single-legged (average of left and right leg) quiet stance trials in the control (white bars), retired (Retired: black bars) and active veteran volleyball athletes’ (Active: gray bars) groups. Data in bars are mean values ± std. error. *Statistically significant group effect (*p* < 0.05). ns: No statistically significant group effect (*p* > 0.05**).**

In the single-legged quiet stance condition, there was no significant main effect of group on either path length (*p *= 0.658, Figure 1A) or anteroposterior CoP range (*p *= 0.813, Figure 1B). A significant main effect of group was found for the mediolateral CoP range (*F* = 26.2; *p *< 0.001) and post-hoc pairwise comparisons showed that the control had a significantly lesser CoP range compared to the Retired and Active veteran volleyball athletes' groups (*p* < 0.001 for both groups) (Figure 1C). No significant differences between the Retired and Active veteran volleyball athletes were found for the mediolateral CoP range (*p* = 1.0, Figure 1C).

**Figure 2:**
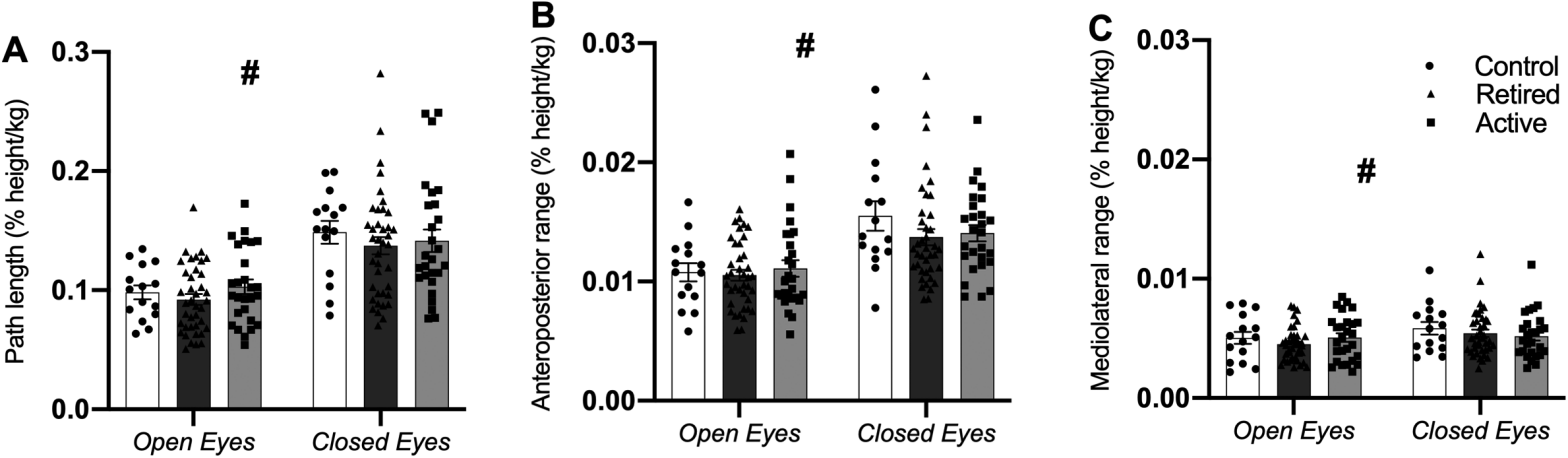
Normalized path length (**A**), CoP range in anteroposterior (**B**) and CoP range in mediolateral direction (**C**) in the two-legged quiet stance trials with eyes open and eyes closed for the control (white bars), retired (retired: black bars) and active veteran volleyball athletes’ (active: gray bars) groups. Data in bars are mean values ± std. error. # Statistically significant vision effect (*p* < 0.05) across groups.

In the two-legged trials, there was no significant main effect of group on either path length (*p *= 0.594, Figure 2A) or the anteroposterior (*p *= 0.531, Figure 2B), or mediolateral CoP range (*p *= 0.646, Figure 2C). There was a statistically significant main effect of vision on path length (*F* = 151.6; *p* < 0.001), the anteroposterior (*F* = 53.8; *p* < 0.001) and mediolateral range of CoP (F = 12.7; *p* < 0.05) (Figure 2). In the eyes-closed condition, path length (Figure 2A), the anteroposterior (Figure 2B) and mediolateral CoP range (Figure 2C) were significantly increased. No significant vision-by-group interaction was found for either path length (*p* = 0.455) or anteroposterior (*p* = 0.379) and mediolateral sway amplitudes (*p* = 0.092) (Figure 2).

There was a statistically significant main effect of group for jump height (F = 19.9; *p* < 0.001), mean (F = 8.7; *p* < 0.001) and maximal power (F = 13.5; *p* < 0.001) in the two-legged CMJ trials. Post-hoc pairwise comparisons showed that the control group had a significantly lower jump height, mean and maximal power compared to both the Retired (Height: *p *< 0.001; Pmean: *p *< 0.01; Pmax: *p *< 0.001) and Active veteran volleyball athletes (Height: *p *< 0.001; Pmean: *p *< 0.001; Pmax: *p *< 0.001) (Table 2). No significant difference between Retired and Active athletes' groups was found for either jump height (*p* = 0.501), or mean (*p* = 0.413) and maximal power (*p* = 0.574, Table 2).

**Table 2:** Vertical jumping performance data for the three groups (mean (SD)).

	Control (*N* = 15)	Retired (*N* = 39)	Active (*N* = 27)
Jump height (cm)*	8.1 (3.3)^a,b^	14.5 (4.7)	15.9 (3.1)
Pmean (Watt/kg)*	13.1 (2.8)^a,b^	15.9 (3.1)	17.0 (2.6)
Pmax (Watt/kg)*	24.2 (5.0)^a,b^	29.9 (4.6)	31.3 (3.6)

*Statistically significant group effect (*p* < 0.05).

^a^ Post hoc statistically significant (*p* < 0.05) differences between control and retired veteran volleyball athletes’ group (Retired).

^b^ Post hoc statistically significant (*p* < 0.05) differences between control and active veteran volleyball athletes’ group (Active).

**Figure 3:**
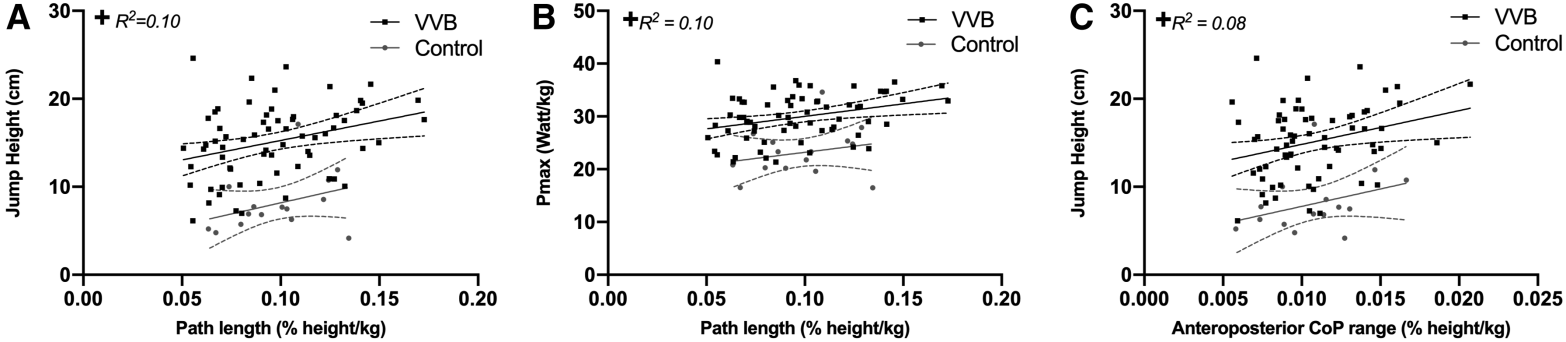
Relationship of normalized CoP path length with jump height (**A**), normalized CoP path length with maximal mechanical power (Pmax) (**B**), and CoP range in anteroposterior direction with jump height (**C**) for the linear regression analysis in the retired and active veteran volleyball athletes’ (VVB) (black circles) and control (gray squares) groups. + Statistically significant associations between two-legged balance and two-legged vertical jumping performance parameters for VVB group (*p* < 0.05).

The linear regression analyses have been performed for the retired and active veteran volleyball athletes as a single group (hereafter VVB group) due to the absence of significant differences in both the two-legged quiet balance and two-legged vertical jumping tasks between these groups. There was a significant positive weak relationship between the CoP path length and CMJ height (*F* = 7.1; *p* = 0.010; *R*^2^: 0.10; RMSE = 3.977, Figure 3A) for the VVB group. Further, a significant positive weak association of the CoP path length and maximal power (*F* = 7.4; *p* = 0.010; *R*^2^: 0.10; RMSE = 4.180, Figure 3B) and a significant positive weak relationship between the anteroposterior sway range and CMJ height (*F* = 5.9; *p* = 0.02; R^2^: 0.08; RMSE = 4.013, Figure 3C) was found for the VVB group. There were no significant associations (*p* > 0.05) observed between the two-legged balance CoP parameters and the vertical jumping performance parameters for the control group (Figure 3).

## 4. Discussion

In this study, we found a greater mediolateral sway range for the female active and retired veteran volleyball athletes compared to a control group of non-athletes during the single-legged task, whereas no differences in postural balance ability were observed among the investigated groups during the two-legged balancing task. Furthermore, we found that both active and retired veteran volleyball athletes had a higher vertical jumping performance compared with non-athletes. Therefore, the first hypothesis concerning a higher balance and jumping performance in the female active and retired veteran volleyball athletes than in the non-athletes is partially supported. Current findings also showed similar postural balance and vertical jumping performance in the active and retired veteran athletes; therefore the second hypothesis regarding a higher performance in the active veteran athletes due to continuous participation in systematic training was rejected. We found a similar increase in CoP amplitude between athletes and non-athletes in the eyes closed condition suggesting a negligible effect of training experience on balance performance deterioration due to visual restriction, thus rejecting our hypothesis. Finally, the weak associations that were found between balance and jumping performance in the active and retired veteran volleyball athletes do not support the last hypothesis regarding a favorable effect of balance on jumping performance.

Postural control and balance is fundamental to activities of daily life. In the sports context, efficient postural balance control helps to achieve skillful execution of sport technique movements (32, 33). In the single-legged static balance task, we found ∼67% higher mediolateral range for the active and retired veteran volleyball athletes compared with the non-athletes. Typically, a low CoP sway amplitude is considered as a positive aspect of postural control, since it is associated with an increased easiness in maintaining postural tasks (34). However, postural responses have been shown to be specific to the context in which the activity is practiced (35, 36) and highly skilled athletes are considered to be able to perform successfully in spite of an increased postural sway (37). In agreement with our findings, elite young adult male and female volleyball athletes were shown to have higher CoP mean sway velocity and frequency in the anteroposterior and mediolateral directions than age-matched, physically active control participants (5, 6). Those authors argued that a low sway amplitude in volleyball athletes could potentially impair the exploratory function of sway (5, 6), with regards to the constant adjustment of the center of mass position in order to achieve optimal body positioning against changing situations on the court. Volleyball training and competition consists of multidirectional sideways locomotion during actions such as defense, blocking, preparation for or landing from a spike jump (16, 38). The greater mediolateral sway behavior that was found for the active and retired veteran athletes compared to the control group might suggest their reliance on a “destabilization–recovery of balance” sequence, where the continuous adjustment of the center of mass position and thus, a greater sway, serves to accelerate the body and provide impetus for movement (39). On the other hand, there were no significant differences in the anteroposterior sway range between the volleyball athletes and the control group during the single-legged stance task. Volleyball athletes have segments of forward and backward locomotion in training or competition, like for example during the preparatory phase before taking off for the spike jump or the jump serve (17, 40). However, the demands for postural control in the forward-backward direction are usually lower compared to the respective ones in sideways direction (41). Therefore, the similar anteroposterior CoP amplitude between the veteran volleyball athletes and the non-athletes might reflect a negligible effect of systematic training on postural responses in the anteroposterior direction.

During the two-legged quiet stance task, postural balance performance was similar among the groups both in the eyes-open and eyes-closed condition. Vision is an important sensory source for postural control since it provides direct information on the position and orientation of the body with respect to the environment (28, 41) and in the absence of visual input, balance performance is deteriorated (42). Athletes in team sports rely strongly on constant visual feedback in order to adequately and timely react to the opponent's actions (9). Furthermore, skilled male volleyball players were found to perform fewer fixations of saccadic eye movements of longer duration, concentrating on the starting and ending points of the ball trajectory, as compared with non-athletes that tended to follow the ball's whole trajectory (43), suggesting specialized utilization of the visual system. However, the expected higher dependence on the visual channel for balance control in the eyes-closed condition was not found for the female veteran volleyball athletes. Our findings agree with earlier reports that showed a similar dependence (i.e., no differences in balance ability) on vision between young adult volleyball players and control participants when the visual input was restricted (9). Postural control depends on the integrated sensory information processing from the visual, vestibular, and proprioceptive systems (28) and in the absence of visual input, proprioception plays a key role in postural control (44) due to the lower threshold for the perception of body sway compared with the visual and vestibular systems (45). Proprioception might therefore be a candidate to explain the lack of the hypothesized aggravation in two-legged balance ability upon restriction of vision in the veteran volleyball athletes.

We found that jump height, mean and maximal mechanical power during the CMJ trials were higher in the active veteran athletes compared to non-athletes. Success in volleyball largely depends on the athlete's ability to jump high since the game's main scoring actions (i.e., attack, serve, block) are performed airborne (12, 15). Considering the continuous participation in systematic training for the active veteran athletes' group, their resulting higher levels of muscle strength and neuromuscular coordination is the most plausible candidate for the observed greater jumping performance compared to the non-athletes. Our findings also revealed that the retired veteran volleyball athletes performed better than the non-athletes in the CMJ task. Earlier studies have reported that former elite male power-trained athletes had a greater vertical jump height (20–22) in comparison with age-matched sedentary men. The retired veteran athletes group was composed of approximately 36% of elite and 64% of top-level female athletes who reported a median lifetime of 7,776 training hours (min-max: 1134–34560 h). The group's prior experience in systematic training, with plyometric training being a critical component of it (18), suggests the positive impact of prolonged systematic training on vertical jumping performance and agrees with previous studies that showed a greater degree of attenuation in the decline of jumping power compared with sedentary healthy participants (21).

It was further found that the retired veteran athletes exhibited similar vertical jumping performance as the active ones. The relatively small difference in performance (i.e., ∼10%) as determined by jump height was also observed in the parameters of the mean and maximal mechanical power (i.e, 7% and 1%), when the difference in body mass was accounted for. Previous *in vivo* investigation on the vastus lateralis muscle intrinsic properties had shown that the crucial parameter contributing to maximal vertical jumping performance was the knee mean mechanical power (46). Taking also into consideration the findings on the similar balance ability between the retired and active veteran athletes, current findings coincide with adaptations in balance, muscle strength and power (20–23, 47) and in the neuromuscular control of vertical jumping tasks (48) previously found in former male athletes resulting from a history of prolonged systematic training. It was expected that the active veteran volleyball athletes would have greater performance compared to the retired athletes as a result of their continuous participation in systematic training; however, the findings did not confirm this hypothesis and might suggest a retention of balance ability and jumping performance in the retired veteran volleyball athletes' group.

Finally, the examination of possible associations between postural balance and vertical jumping performance yielded weak associations (i.e., average *R*^2^ = 9.5%) between CoP amplitude and the jumping performance parameters for the female veteran volleyball athletes as a single group. Even though, the findings indicate that balance ability has a negligible effect on jumping performance in active and retired veteran volleyball athletes, both groups of athletes would probably benefit from adequate postural balance control since it would provide a stable upper body being in tune with the lower body movements, thus generating favourable conditions for the vertical jumping task (49). On the other hand, the lack of associations of balance ability with vertical jumping performance in the non-athletes is most likely related to the absence of systematic physical activity and its role in preserving a satisfactory level of physical conditioning and motor skills in middle-aged participants.

This study has some limitations that should be addressed. The non-athletes were sedentary participants, indicating generally lower physical activity levels compared with the active veteran volleyball athletes. Their sedentary level was determined on the basis of a 5-year cut-off period (25), which was considered as an adequate time period to “wash out” any possible effect of past regular physical activity. The same bias could exist between non-athletes and the retired veteran athletes, who preserved a certain level of fitness resulting from their recreational engagement in physical activities. Further, both the active and retired veteran volleyball athletes were taller and heavier than the non-athletes and thus, we normalized the assessment of postural balance parameters to body height and body mass excluding the bias due to these two parameters; however, the bias of the different physical activity levels remained in this study. We also sought to examine the contribution of the visual channel on balance performance in the veteran volleyball athletes and to achieve this, the CoP outcomes were considered for 15 s in each of the two visual conditions (eyes open and eyes closed). It has been suggested that during tasks involving vision occlusion transient responses within the CoP data may occur and, thus longer trials may improve the reliability of quiet stance postural control assessments (50). However, with a longer duration per trial, fatigue would accumulate, most probably for the non-athletes, and could introduce bias into the comparisons among the groups.

In conclusion, we found higher balance ability and vertical jumping performance in female active and retired veteran volleyball athletes compared to a control group of non-athletes. The greater balance and vertical jumping performance of the retired veteran volleyball athletes compared to non-athletes suggest the positive impact of prior systematic history of sport participation on the preservation of an enhanced physical activity level. The restriction of visual information decreased postural balance performance similarly in all three investigated groups, suggesting that the veteran volleyball athletes did not rely solely on vision for postural control during a two-legged stance task. Static two-legged balance ability is not associated with performance in the two-legged countermovement jump in female active and retired veteran volleyball athletes. Volleyball can be recommended as a beneficial exercise modality with the potential to counter the age-related impairments in physical conditioning.

## Data Availability

The raw data supporting the conclusions of this article will be made available by the authors, without undue reservation.

## References

[1] Fuchs PX Menzel HK Guidotti F Bell J von Duvillard SP Wagner H. Spike jump biomechanics in male versus female elite volleyball players. J Sports Sci. (2019) 37:2411–19. 10.1080/02640414.2019.1639437 31280702

[2] Palao JM Santos JA Ureña A. Effect of team level on skill performance in volleyball. Int J Perform Anal Sport. (2004) 4:50–60. 10.1080/24748668.2004.11868304

[3] Ciapponi T.M. Mclaughlin E.J., and Hudson J.L. (1996). The volleyball approach: an exploration of balance, 282–285. Abstract retrieved from abstracts in 13th international symposium on biomechanics in sports. Thunder Bay, oN: Lakehead University School of Kinesiology

[4] Marquez WQ Masumura M Ae M. Spike-landing motion of elite male volleyball players during official games. Int J Sport Health Sc. (2011) 9:82–90. 10.5432/ijshs.20100015

[5] Borzucka D Kręcisz K Rektor Z Kuczyński M. Differences in static postural control between top level male volleyball players and non-athletes. Sci Rep. (2020) 10:19334. 10.1038/s41598-020-76390-x 33168913 PMC7653955

[6] Borzucka D Kręcisz K Rektor Z Kuczyński M. Postural control in top-level female volleyball players. BMC Sports Sci Med Rehabil. (2020) 12:65. 10.1186/s13102-020-00213-9 33101691 PMC7576872

[7] Forthomme B Croisier JL Ciccarone G Crielaard JM Cloes M. Factors correlated with volleyball spike velocity. Am J Sports Med. (2005) 33:1513–19. 10.1177/0363546505274935 16009986

[8] Kuczyński M Rektor Z Borzucka D. Postural control in quiet stance in the second league male volleyball players. Hum Mov. (2009) 10:12–5. 10.2478/v10038-008-0025-4

[9] Agostini V Chiaramello E Canavese L Bredariol C Knaflitz M. Postural sway in volleyball players. Hum Mov Sci. (2013) 32:445–56. 10.1016/j.humov.2013.01.002 23628360

[10] Sarto F Grigoletto D Baggio E Paoli A Marcolin G. Do lower limb previous injuries affect balance performance? An observational study in volleyball players. Phys Ther Sport. (2019) 37:49–53. 10.1016/j.ptsp.2019.02.009 30851569

[11] Polglaze T Dawson B. The physiological requirements of the positions in state league volleyball. Sports Coach. (1992) 15:32–7.

[12] Skazalski C Whiteley R Bahr R. High jump demands in professional volleyball—large variability exists between players and player positions. Scand J Med Sci Sports. (2018) 28:2293–98. 10.1111/sms.13255 29956376

[13] Lidor R Ziv G. Physical and physiological attributes of female volleyball players-a review. J Strength Cond Res. (2010) 24:1963–73. 10.1519/JSC.0b013e3181ddf835 20543736

[14] Ziv G Lidor R. Vertical jump in female and male volleyball players: a review of observational and experimental studies. Scand J Med Sci Sports. (2010) 20:556–67. 10.1111/j.1600-0838.2009.01083.x 20459471

[15] Berriel GP Schons P Costa RR Oses VHS Fischer G Pantoja PD Correlations between jump performance in block and attack and the performance in official games, squat jumps, and countermovement jumps of professional volleyball players. J Strength Cond Res. (2021) 35:S64–9. 10.1519/JSC.0000000000003858 33337704

[16] Sheppard JM Gabbett TJ Stanganelli LC. An analysis of playing positions in elite men's Volleyball: considerations for competition demands and physiologic characteristics. J Strength Cond Res. (2009) 23:1858–66. 10.1519/JSC.0b013e3181b45c6a 19675472

[17] Ikeda Y Sasaki Y Hamano R. Factors influencing spike jump height in female college volleyball players. J Strength Cond Res. (2018) 32:267–73. 10.1519/JSC.0000000000002191 28902117

[18] Ramirez-Campillo R Andrade DC Nikolaidis PT Moran J Clemente FM Chaabene H Effects of plyometric jump training on vertical jump height of volleyball players: a systematic review with meta-analysis of randomized-controlled trial. J Sports Sci Med. (2020) 19:489–99. 32874101 PMC7429440

[19] International Veteran Volleyball Association. https://www.ivva.eu/ivva-indoor-volleyball-tournament/(Accessed July 15, 2022).

[20] Kettunen JA Kujala UM Räty H Sarna S. Jumping height in former elite athletes. Eur J Appl Physiol Occup Physiol. (1999) 79:197–201. 10.1007/s004210050495 10029342

[21] Manderoos S Wasenius N Laine MK Kujala UM Mälkiä E Kaprio J Mobility and muscle strength in male former elite endurance and power athletes aged 66-91 years. Scand J Med Sci Sports. (2017) 27:1283–91. 10.1111/sms.12775 27704644

[22] Räty HP Impivaara O Karppi SL. Dynamic balance in former elite male athletes and in community control subjects. Scand J Med Sci Sports. (2002) 12:111–16. 10.1034/j.1600-0838.2002.120208.x 12121429

[23] Sipilä S Viitasalo J Era P Suominen H. Muscle strength in male athletes aged 70-81 years and a population sample. Eur J Appl Physiol Occup Physiol. (1991) 63:399–403. 10.1007/BF00364469 1773819

[24] Brauer SG Neros C Woollacott M. Balance control in the elderly: do masters athletes show more efficient balance responses than healthy older adults? Aging Clin Exp Res. (2008) 20:406–11. 10.1007/BF03325145 19039281

[25] Bulbulian R Hargan ML. The effect of activity history and current activity on static and dynamic postural balance in older adults. Physiol Behav. (2000) 70:319–25. 10.1016/s0031-9384(00)00272-9 11006430

[26] Lee C Fleming N Donne B. Comparison of balance variables across active and retired athletes and age matched controls. Int J Exerc Sci. (2021) 14:76–92. 34055155 10.70252/YPEF5513 PMC8136550

[27] Nikolaidou ME Schroll A Sotiropoulos K Drikos S Barzouka K Arampatzis A. Postural stability performance in female veteran volleyball players, 87-91. Short paper retrieved from the Proceedings of the 29th International on Physical Education & Sport Science, Komotini, 14-16 May 2021 (2021).

[28] Horak FB Shupert CL Mirka A. Components of postural dyscontrol in the elderly: a review. Neurobiol Aging. (1989) 10:727–38. 10.1016/0197-4580(89)90010-9 2697808

[29] Prieto TE Myklebust JB Hoffmann RG Lovett EG Myklebust BM. Measures of postural steadiness: differences between healthy young and elderly adults. IEEE Trans Biomed Eng. (1996) 43:956–66. 10.1109/10.532130 9214811

[30] Bryant EC Trew ME Bruce AM Kuisma RME Smith AW. Gender differences in balance performance at the time of retirement. Clin Biomech. (2005) 20:330–35. 10.1016/j.clinbiomech.2004.11.006 15698707

[31] Hue O Simoneau M Marcotte J Berrigan F Doré J Marceau P Body weight is a strong predictor of postural stability. Gait Posture. (2007) 26:32–8. 10.1016/j.gaitpost.2006.07.005 16931018

[32] Marcolin G Supej M Paillard T. Editorial: postural balance control in sport and exercise. Front Physiol. (2022) 13:961442. 10.3389/fphys.2022.961442 35903066 PMC9315383

[33] Paillard T. Plasticity of the postural function to sport and/or motor experience. Neurosci Biobehav Rev. (2017) 72:129–52. 10.1016/j.neubiorev.2016.11.015 27894829

[34] Horak FB. Postural orientation and equilibrium: what do we need to know about neural control of balance to prevent falls? Age Ageing. (2006) 35(Suppl 2):ii7–ii11. 10.1093/ageing/afl077 16926210

[35] Paillard T Noé F. Effect of expertise and visual contribution on postural control in soccer. Scand J Med Sci Sports. (2006) 16:345–48. 10.1111/j.1600-0838.2005.00502.x 16978254

[36] Zemková E. Sport-specific balance. Sports Med. (2014) 44:579–90. 10.1007/s40279-013-0130-1 24293269

[37] Paillard T. Relationship between sport expertise and postural skills. Front Psychol. (2019) 10:1428. 10.3389/fpsyg.2019.01428 31293483 PMC6603331

[38] Sheppard JM Gabbett T Taylor KL Dorman J Lebedew AJ Borgeaud R. Development of a repeated-effort test for elite men's Volleyball. Int J Sports Physiol Perform. (2007) 2:292–304. 10.1123/ijspp.2.3.292 19168929

[39] Le Mouel C Brette R. Mobility as the purpose of postural control. Front Comput Neurosci. (2017) 11:67. 10.3389/fncom.2017.00067 28798679 PMC5529402

[40] Wagner H Tilp M von Duvillard SP Mueller E. Kinematic analysis of volleyball spike jump. Int J Sports Med. (2009) 30:760–65. 10.1055/s-0029-1224177 19585402

[41] Winter D. Human balance and posture control during standing and walking. Gait Posture. (1995) 3:193–214. 10.1016/0966-6362(96)82849-9

[42] Paulus WM Straube A Brandt T. Visual stabilization of posture. Physiological stimulus characteristics and clinical aspects. Brain. (1984) 107:1143–63. 10.1093/brain/107.4.1143 6509312

[43] Piras A Lobietti R Squatrito S. A study of saccadic eye movement dynamics in volleyball: comparison between athletes and non-athletes. J Sports Med Phys Fitness. (2010) 50:99–108. 20308980

[44] Wiesmeier IK Dalin D Maurer C. Elderly use proprioception rather than visual and vestibular cues for postural motor control. Front Aging Neurosci. (2015) 7:97. 10.3389/fnagi.2015.00097 26157386 PMC4477145

[45] Speers RA Kuo AD Horak FB. Contributions of altered sensation and feedback responses to changes in coordination of postural control due to aging. Gait Posture. (2002) 16:20–30. 10.1016/s0966-6362(02)00003-6 12127183

[46] Nikolaidou ME Marzilger R Bohm S Mersmann F Arampatzis A. Operating length and velocity of human M. vastus lateralis fascicles during vertical jumping. R Soc Open Sci (2017) 4:170185. dx. 10.1098/rsos.170185 28573027 PMC5451828

[47] Izquierdo M Häkkinen K Gonzalez-Badillo JJ Ibáñez J Gorostiaga EM. Effects of long-term training specificity on maximal strength and power of the upper and lower extremities in athletes from different sports. Eur J Appl Physiol. (2002) 87:264–71. 10.1007/s00421-002-0628-y 12111288

[48] Masci I Vannozzi G Gizzi L Bellotti P Felici F. Neuromechanical evidence of improved neuromuscular control around knee joint in volleyball players. Eur J Appl Physiol. (2010) 108:443–50. 10.1007/s00421-009-1226-z 19826834

[49] Butcher SJ Craven BR Chilibeck PD Spink KS Grona SL Sprigings EJ. The effect of trunk stability training on vertical takeoff velocity. J Orthop Sports Phys Ther. (2007) 37:223–31. 10.2519/jospt.2007.2331 17549950

[50] Reed CA Chaudhari AMW Worthen-Chaudhari LC Bigelow KE Monfort SM. A new perspective on transient characteristics of quiet stance postural control. PLoS ONE. (2020) 15:e0237246. 10.1371/journal.pone.0237246 32776952 PMC7416949

